# Advances in Energy Harvesters/Nanogenerators and Self-Powered Sensors II

**DOI:** 10.3390/nano14010121

**Published:** 2024-01-04

**Authors:** Jianxiong Zhu, Qiongfeng Shi

**Affiliations:** 1 School of Mechanical Engineering, Southeast University, Nanjing 211189, China; 2 Engineering Research Center of New Light Sources Technology and Equipment, Ministry of Education, Nanjing 211189, China; 3 Interdisciplinary Research Center, School of Electronic Science and Engineering, Southeast University, Nanjing 211189, China

The Internet of Things (IoT) has become a focal point in the realm of information technology and has facilitated the interconnectedness and communication of various objects, such as devices and sensors in smart cities, intelligent transportation, industrial automation, agriculture, healthcare, etc. To meet the extensive and widely dispersed power requirements essential for IoT nodes, self-sustained systems powered by energy harvesting technologies, including but not limited to piezoelectric, triboelectric, electromagnetic, thermoelectric, pyroelectric, and photovoltaic methods, offer the potential for substantial benefits. Research groups around the globe have extensively explored these technologies, resulting in innovative energy harvesters and self-powered devices that enable the realization of self-sustained and functional systems and open up a myriad of promising applications in the new era. For example, these applications span wearable sensors, medical devices, home systems, and entertainment. The ultimate goal is to enhance the convenience of human life through the integration of these technologies in various aspects of daily living. Meanwhile, the microfabrication techniques employed in creating these advanced sensing and smart systems have progressively evolved to become more sustainable and robust.

This Special Issue, “Advances in Energy Harvesters/Nanogenerators and Self-Powered Sensors II”, aims to present the latest breakthroughs and contributions in the field of micro/nanogenerators, energy harvesters, self-powered sensors, and their system applications. It features research papers and review articles that introduce the design, manufacture, integration, characterization, and use of energy harvesters, self-powered sensors, nanogenerators, and systems.

It also includes ten research articles and one review article from researchers across the world that explore technological innovations in the field of energy harvesting and self-powered sensing. Zhao et al. [1] introduce a versatile, self-sustaining displacement sensor using the triboelectric effect. It is designed for seamless integration in linear feed systems, enabling the precise detection of displacements within a broad range. It also possesses the capability for real-time velocity detection in linear feed systems, achieving an impressive accuracy rate of below 0.5%. To precisely measure the occurrence of minor tangential slips at contact interfaces during cyclic loadings in a machine system, Zhao et al. [2] developed a self-powered displacement sensor utilizing a triboelectric nanogenerator. This sensor incorporates a 100 nm thick BaTiO_3_ thin film positioned between the Kapton triboelectric layer and the Cu electrode. Then, Yoon et al. [3] introduce the design of an Ag:AZO electrode for a self-powered solar-blind ultraviolet (UV) photodetector utilizing the Ag_2_O/β-Ga_2_O_3_ heterojunction. The 20 nm Ag:AZO electrode with the lowest surface roughness had an on/off ratio of 2.01 × 10^8^, and its responsivity and detectivity were 56 mA/W and 6.99 × 10^11^, respectively, when the optoelectronic properties were gauged under 254 nm UV-C light. Kim et al. [4] present a polymethyl methacrylate passivation layer on the cathode side to enhance the efficiency and stability of a self-powered photodiode using CH_3_NH_3_PbI_3_ perovskite. When compared with a normal MAPbI_3_ photodiode without a PMMA layer, this device showed a much higher specific detectivity value (~1.07 × 10^12^) in the absence of external bias.

In terms of energy harvesting from ambient sources, Li et al. [5] introduce an innovative solid–liquid triboelectric nanogenerator using vortex-induced resonance to efficiently harvest ocean energy over extended periods. This device captures energy through the resonance between vortex-induced vibrations of a cylinder and the relative movements of solid–liquid friction pairs within the cylinder. Then, Mao et al. [6] present a technology to enhance the triboelectric achievement of a PDMS-based triboelectric nanogenerator through adding nanostructured cadmium sulfide. The N-Cds/PDMS substrates demonstrated superior triboelectric performance with a high short-circuit current and open-circuit voltage. Meanwhile, Duque et al. [7] designed, manufactured, and electrically evaluated a piezoelectric resonant energy harvester utilizing 3D printing. This device demonstrated a maximum output power of 1.46 mW under optimal conditions, i.e., 4 MΩ load impedance and 1 g acceleration. To take advantage of acoustic waves of lower frequency in marine and industrial environments, Xiao et al. [8] present a novel acoustic triboelectric nanogenerator comprising a quarter-wavelength resonant tube, an FEP membrane, an evenly perforated aluminum film, and a conductive coating of carbon nanotube. To efficiently manufacture Sb_2_Se_3_/CdS-based solar cells for solar energy harvesting, Kumari et al. [9] developed a device designed for easy reproduction in any laboratory through the utilization of the thermal evaporation technique. Wong et al. [10] utilized a BN/GaN layered composite along with aluminum to fabricate Schottky diodes with the goal of harvesting high-frequency wireless energy. Through the adjustment of the induced electric field at the interface, they discovered that the dielectric constant of the boron nitride monolayer atop the aluminum monolayer was as low as 1.5, indicating its potential for capturing high-band 5G signals.

Furthermore, for readers interested in an overview of triboelectric and piezoelectric nanogenerators, the review article by Delgado-Alvarado et al. [11] report the recent advancements in piezoelectric and triboelectric nanogenerators designed for self-powered monitoring healthcare devices. The article covers the working principles, fabrication processes, and materials of various nanogenerators. Discussions on the electrical behavior of both piezoelectric and triboelectric nanogenerators are provided. Additionally, they explored signal processing components and diverse packaging types for these nanogenerators. This review thoroughly summarizes the design, materials, and electromechanical performance of nanogenerators designed for biomechanical energy harvesting, emphasizing their potential for various medical applications.

Finally, we want to extend our gratitude to all the authors and reviewers who contributed to this Special Issue. We hope that the articles featured in this Special Issue are interesting and helpful for the broad readerships of *Nanomaterials* and can inspire new innovations in the current state of the art regarding energy harvesting, advanced sensing, and smart systems.
